# Intramedullary parasite eggs, latent for three decades, mimicking acute transverse myelitis

**DOI:** 10.1186/s12879-021-07013-7

**Published:** 2022-01-04

**Authors:** Hyo-jeong Kim, Se-Hoon Kim, Hoi-seon Jeong, Bum-Joon Kim

**Affiliations:** 1 Department of Neurosurgery, Ansan Hospital, Korea University College of Medicine, 123 Jeokgeum-ro, Danwon-gu, Ansan, Gyeonggi-do 15355 Republic of Korea; 2 Department of Pathology, Ansan Hospital, Korea University College of Medicine, Ansan, Republic of Korea

**Keywords:** Central nervous system parasitic infections, Parasite eggs, Neurocysticercosis, Brown-Sequard syndrome, Spinal cord neoplasm, Acute transverse myelitis

## Abstract

**Background:**

Intramedullary parasitic infection is extremely uncommon, and clinical presentation of Brown-Sequard syndrome is even rarer.

**Case presentation:**

The authors report a case involving a 57-year-old woman with Brown-Sequard syndrome, in whom magnetic resonance imaging and clinical and epidemiological features were similar to those of acute transverse myelitis. Myelotomy suggested inflammation caused by latent parasite eggs in the spinal cord. Antiparasitic and steroid therapies were administered postoperatively. To the author’s knowledge, this is the first report to describe a surgical experience for *Taenia solium* eggs in the spinal cord.

**Conclusion:**

Intramedullary parasitic infection is a diagnostic challenge that requires careful discrimination from other diseases. If parasite infection is suspected in a progressively deteriorating patient, myelotomy should be considered for rapid and accurate treatment.

## Background

Parasitic infections, which are transmitted by various routes, are endemic in countries with poor sanitary conditions and rarely occur in developed countries. Among parasitic infections, those of the central nervous system (CNS) are generally devastating, with high mortality and morbidity rates [1]. However, some of these infections can be controlled by the host immune response and may persist as chronic infections for many years [2].

Spinal involvement of neurocysticercosis (NCC), one of the most common parasitic infections of the CNS, accounts for only 2.7% of all patients with NCC [3]. While it is important to treat spinal parasite infections, it is critical to properly differentiate parasites from other probable causes, including acute transverse myelitis (ATM), intramedullary cystic lesions (arachnoid cyst, ependymal cyst, neurenteric cyst, sarcoidosis), intramedullary tumor, multiple sclerosis, tuberous sclerosis, and infections (pyogenic abscess, tuberculoma, and toxoplasmosis) [4]. Furthermore, the differential diagnosis between parasites is also important because the treatments differ among diseases.

Herein, we report a rare case of intramedullary parasite infection presenting with clinical features of Brown-Sequard syndrome mimicking ATM.

## Case report

**Fig. 1:**
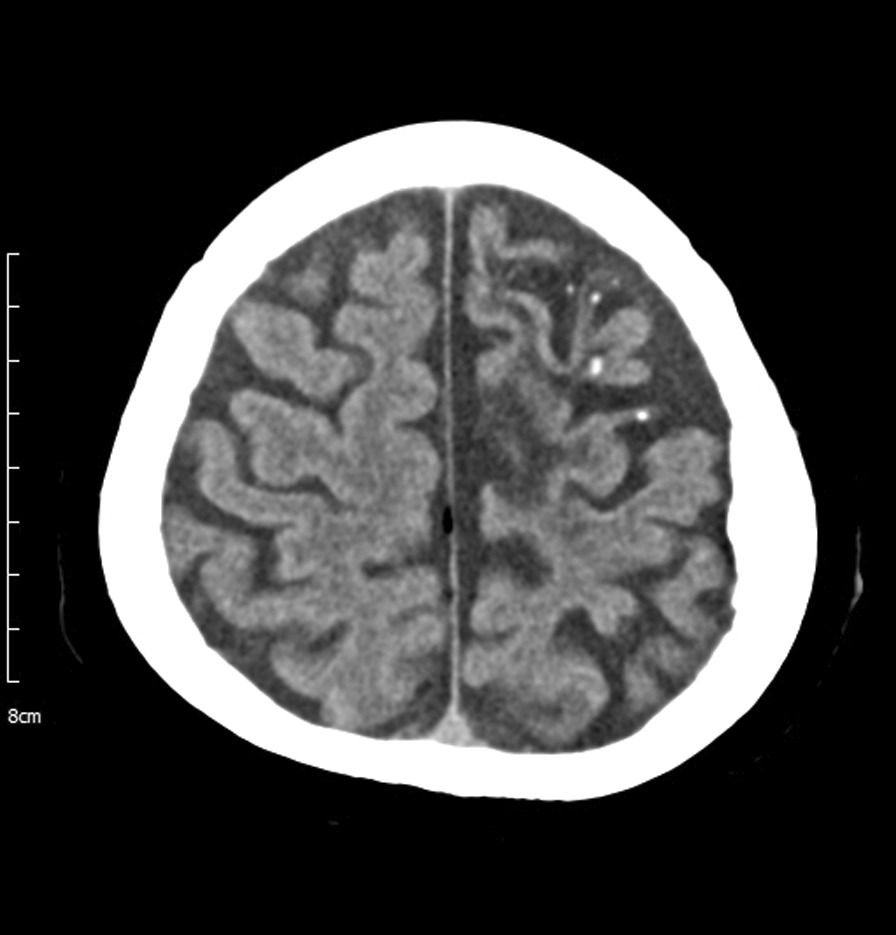
Brain computed tomography of the patient

A 57-year-old Korean woman with a 10-year history of limping of the right leg due to the sequelae of neurocysticercosis, visited our outpatient clinic with complaints of progressive weakness in her right leg and numbness in her left leg. She reported that, although she was usually able to walk independently, the paralysis of her right leg had rapidly progressed over the previous month ago and she had been completely wheelchair-bound for the past month. She had no history of trauma, and she mentioned that the weakness of her right leg had progressed insidiously. She had been taking valproic acid since visiting the National Medical Center for seizures 30 years prior. She showed no definite side effects of this drug such as asthenia, tremor, dizziness, or drowsiness. At that time, NCC was suspected based on multiple calcified lesions in the left frontoparietal subcortical area on brain computed tomography (CT) (Fig. 1); however, she did not take anti-parasite medication because there was no viable cyst. She grew up in a rural area with rice fields and livestock consisting mainly of pigs. Although her family sometimes ate raw meat, the patient indicated that she had not enjoyed eating meat since childhood.

In the outpatient clinic, she was afebrile with stable vital signs. The patient was alert and exhibited no cognitive dysfunction. Her cognitive function was assessed using the Korean version of the Mini-Mental State Exam, and her score, higher than 24, was within the normal range. Neurological examination of the cranial nerves and upper extremities revealed no definite abnormalities. However, her right lower extremity exhibited severe (grade 0–1) weakness in all muscle groups with hyper-reflexia. In addition, her right lower extremity showed a decreased fine touch and vibration sensation. While the left lower extremity exhibited a normal motor grade, the patient reported decreased pain and temperature sensations on her left side below the T4 dermatome level. She was admitted immediately, and the medical team prepared for the possibility of emergency surgery while performing radiological and laboratory examinations. The next day, her right leg weakness had spontaneously improved to grade 3. Thereafter, her symptoms repeatedly increased and decreased during hospitalization.

**Fig. 2:**
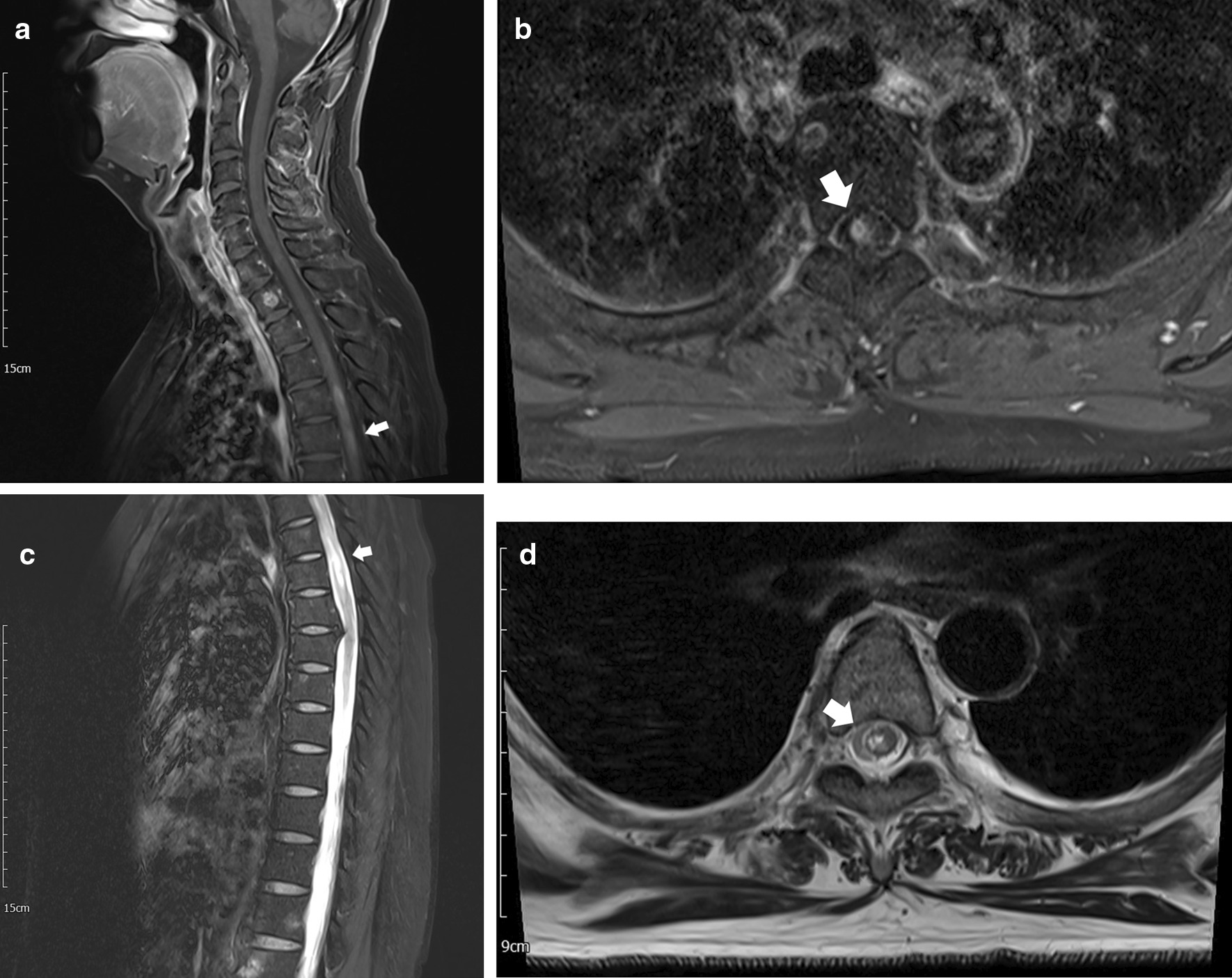
Preoperative magnetic resonance images of the patient

Contrast-enhanced magnetic resonance imaging (MRI) revealed a small ring-enhancing lesion in the right anterior aspect of her spinal cord at the level of T4, with extensive cord edema from T2 to upper T6 (Fig. 2). Laboratory findings including leukocyte count, electrolytes, and tumor markers (including markers for tumors that metastasize easily to the spine, such as carcinoembryonic antigen, cancer antigen 15–3, squamous cell carcinoma antigen, and alpha fetoprotein) were normal. Serum and cerebrospinal fluid (CSF) enzyme-linked immunosorbent assay (ELISA) for *Taenia solium* and *Clonorchis sinensis* antibodies were within the normal ranges. However, CSF analysis revealed increased leukocyte count (corrected count, 40/μL), which was lymphocyte dominant. In addition, the CSF was positive for aquaporin-4 antibody.

Inter-departmental discussions were conducted with neurology, radiology, and infectious diseases. Imaging findings in this patient did not reveal the typical scolex on MRI, and the aquaporin-4 antibody positivity could not exclude a diagnosis of ATM (especially neuromyelitis optica). The possibility of an intramedullary tumor also could not be excluded. Considering the ring-shaped lesion shown on MRI, her previous history of NCC, clinical presentation, and lymphocytic pleocytosis in the CSF, a spinal parasite infection (specifically NCC) was suspected for which she underwent an open biopsy.

**Fig. 3:**
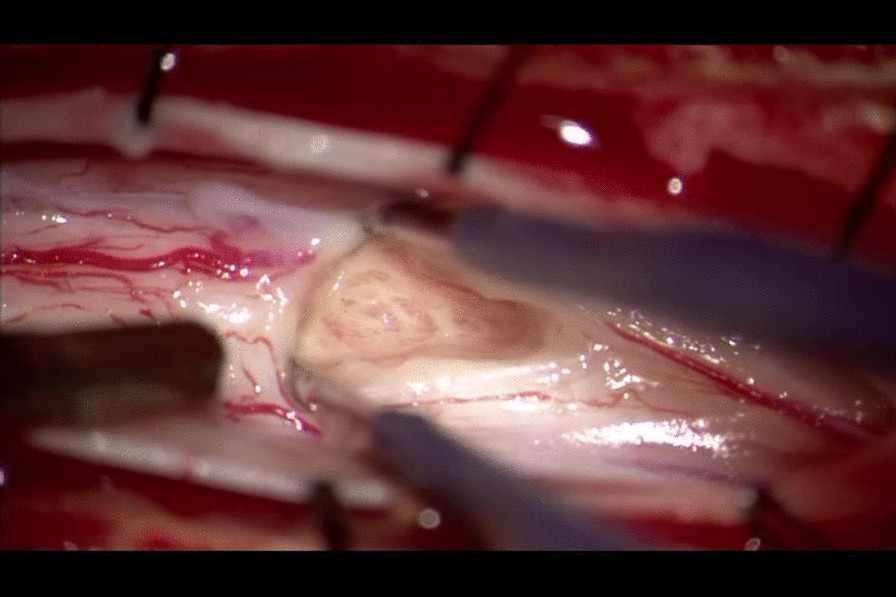
Intraoperative photography. Myelotomy T3–T5, open biopsy

**Fig. 4:**
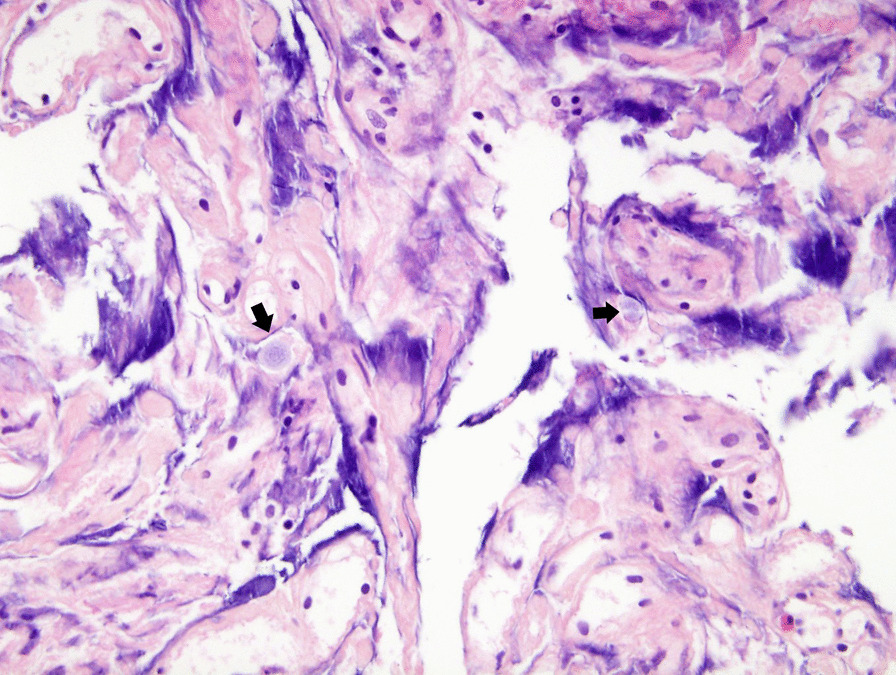
Photomicrographs of the histopathological specimen. Tissue from the ring-enhancing lesion revealed on magnetic resonance imaging was obtained by myelotomy and open biopsy. Tissue from the spinal cord exhibited glial tissue with diffuse infiltration of mononuclear inflammatory cells. At high magnification, round to oval-shape foreign bodies, presumed to be parasite eggs, were observed (hematoxylin and eosin stain, original magnification ×400)

The patient underwent surgery under general anesthesia in the prone position. The T3, T4, and T5 lamina were removed en bloc using an ultrasonic osteotome. When the dura was opened, the spinal cord exhibited no swelling or exophytic lesions and was essentially normal. Myelotomy along the posterior median sulcus revealed spiderweb-like structures and gliotic changes around the central canal but no definite scolex or tumorous lesions (Fig. 3). Biopsy specimens were collected from the area suspected to be the enhancing nodule on preoperative MRI. After dural re-sealing, the laminar flap was secured using mini-plates. Histopathological examination revealed bluish ovoid foreign bodies, presumed to be parasite eggs, among glial tissues with the diffuse infiltration of mononuclear inflammatory cells (Fig. 4). The 30–40-µm spherical eggs containing hook-like structures seemed compatible with the properties of eggs of the Taenia species [5].

After surgery, the patient exhibited immediate improvement in weakness to a grade 4 level and the tingling symptom in her left leg subsided. On postoperative MRI, the ring-enhancing lesion had disappeared, and the cord edema had somewhat improved. Postoperatively, intravenous dexamethasone 5 mg was administered four times a day at 6-h intervals (0.30 mg/kg/day) for a total of 4 days by reducing the dose by 5 mg per day. To reduce perilesional edema, oral antiparasitic drugs (albendazole [800 mg] once daily and praziquantel [1200 mg] four times daily) were administered for 14 days from the day after starting steroid administration. The patient was later referred for rehabilitation and was able to walk independently 6 months later.

## Discussion and conclusions

Parasitic infections occur mainly by chance; however, many, including *T. solium*, occur following the ingestion of food contaminated with parasitic eggs from human or animal feces [2]. Hence, they have a worldwide distribution but are particularly prevalent in low-income countries lacking sanitization and hygiene [6]. Parasitic infections of the CNS are known for their high mortality and morbidity rates [1]. However, the symptoms of CNS invasion of parasites depend on the number of parasites and location, and the host immune response [2]. Clinical symptoms of spinal parasitic infections include simple back pain, numbness, weakness, bowel/bladder incontinence or, rarely, Brown-Sequard syndrome [7].

While various parasites can cause spinal infection, *T. solium*, Schistosoma species, *Echinococcus granulosus*, and *Toxoplasma gondii* are most frequently reported [7]. Considering that South Korea is not endemic for Schistosoma species or *E. granulosus*, and the intraparenchymal calcification of the patient’s brain, it was presumed to be *T. solium* eggs. Although *T. solium* is the most common parasite to induce CNS infection [8], spinal cysticercosis has a very low prevalence, accounting for only 1–5% of patients with NCC [9], and the prevalence of the intramedullary form of NCC is even lower, with only 55 cases reported up to 2014 [4].

Neuroimaging, such as CT and MRI, reveal the morphology and localization of cysts, burden of infection, cyst stages, and surrounding inflammation [10, 11]. Cysticercosis, the most common CNS-affecting parasitic infection, involves four stages that can be identified using MRI: first (vesicular) stage, cyst and scolex without enhancement; second (colloidal) stage, ring enhancement with edema; third (granular nodular degeneration) stage, decreased enhancement and edema; and fourth (involution) stage, obvious calcification [10]. However, because parasite degeneration is a continuum rather than a staged process, neuroimaging is not always precise [11].

Histopathological examination using open biopsy of the brain or spinal cord, which appears as a scolex (spiral canal and rostellum) or three-layered membrane, can be helpful for the diagnosis of NCC rather than other parasitic infections [12]. However, in the second to fourth stages of NCC (colloidal to involution stage), this typical feature is absent [12]. CSF results can be helpful in differential diagnosis but are not pathognomonic because moderate lymphocytic pleocytosis, variable eosinophilic pleocytosis, elevated protein, and low or normal glucose levels may occur [13]. In addition, ELISA is a limited method for diagnosis because it has poor sensitivity for parenchymal NCC and requires high levels of circulating antigens and antibodies to produce a positive result [10].

In this case, making the distinction from ATM was quite difficult. MRI revealed diffuse cord edema from T2 to T6 with subtle contrast enhancement, and the CSF study was positive for aquaporin-4 antibody, a hallmark feature of the diagnosis of neuromyelitis optica [14]. However, unlike ATM, which usually exhibits a short onset to symptom nadir time (within 1 week), or tumors whose symptoms gradually worsen, the symptoms in our patient had repeatedly increased and decreased for > 1 month [15]. In addition, the patient did showed no characteristic symptoms of anti-aquaporin 4 antibody–related disease, such as a visual field defect, decreased visual acuity, or loss of color vision.

Although not standardized, the treatment of parasitic CNS infection(s) includes both pharmaceutical (antiparasitic and anti-inflammatory drugs) and surgical measures. Although older studies reported high mortality and morbidity rates for surgery, advances in microsurgery techniques in recent years have enabled surgery to be performed without major neurological deficits [16]. We suggest that surgery is indicated for the relief of acute symptoms caused by the mass effect or for patients with progressive neurological dysfunction. Lesion location should also be considered in the decision to perform surgery. Pharmaceutical treatment (antiparasitic and anti-inflammatory drugs) should be considered for diagnostic and therapeutic purposes when an infection is suspected and there are no progressive neurological defects. Intramedullary parasitic infections are very rare and difficult to diagnose because they are easily confused with other diseases, including intramedullary tumors and ATM. If a parasitic infection is suspected and the patient experiences progressive deterioration, invasive myelotomy should be considered for rapid and accurate treatment; otherwise, patients can be successfully treated using a combination of spinal cord decompression and medication.

## Data Availability

All the information supporting our conclusions are included in the manuscript. There are no datasets related to this case report.

## Abbreviations

CNS: Central nervous system
NCC: Neurocysticercosis
ATM: Acute transverse myelitis
CT: Computed tomography
MRI: Magnetic resonance imaging
CSF: Cerebrospinal fluid
ELISA: Enzyme-linked immunosorbent assay

## References

[1] Mallewa M, Wilmshurst JM (2014). Overview of the effect and epidemiology of parasitic central nervous system infections in African children. Semin Pediatr Neurol.

[2] Dzikowiec M, Góralska K, Błaszkowska J (2017). Neuroinvasions caused by parasites. Ann Parasitol.

[3] Siddharth Shah SD (2017). Cysticercosis of the spine: a review. Arch Parasitol.

[4] Chaurasia RN, Mishra VN, Jaiswal S (2015). Spinal cysticercosis: an unusual presentation. BMJ Case Rep.

[5] Del Brutto OH, García HH. *Taenia solium*: biological characteristics and life cycle. In: Cysticercosis of the human nervous system. Berlin, Heidelberg: Springer; 2014. 10.1007/978-3-642-39022-7_2.

[6] Schnepper GD, Johnson WD. Recurrent spinal hydatidosis in North America. Case report and review of the literature. Neurosurg Focus. 2004;17:E8. 10.3171/foc.2004.17.6.8 15636578

[7] Majmundar N, Patel PD, Dodson V, Tran A, Goldstein I, Assina R (2019). Parasitic infections of the spine: case series and review of the literature. Neurosurg Focus.

[8] Spallone A, Woroch L, Sweeney K, Seidman R, Marcos LA (2020). The burden of neurocysticercosis at a single New York hospital. J Pathog.

[9] De Souza QL, Filho AP, Callegaro D, De Faria LL (1975). Intramedullary cysticercosis. Case report, literature review and comments on pathogenesis. Case report. J Neurol Sci.

[10] Garcia HH, Nash TE, Del Brutto OH (2014). Clinical symptoms, diagnosis, and treatment of neurocysticercosis. Lancet Neurol.

[11] Mathuriya SN, Khosla VK, Vasishta RK, Tewari MK, Pathak A, Prabhakar S (2001). Intramedullary cysticercosis: MRI diagnosis. Neurol India.

[12] Del Brutto OH, Nash TE, White AC, Rajshekhar V, Wilkins PP, Singh G, Vasquez CM, Salgado P, Gilman RH, Garcia HH (2017). Revised diagnostic criteria for neurocysticercosis. J Neurol Sci.

[13] Ahmad FU, Sharma BS (2007). Treatment of intramedullary spinal cysticercosis: report of 2 cases and review of literature. Surg Neurol.

[14] Waters P, Jarius S, Littleton E, Leite MI, Jacob S, Gray B, Geraldes R, Vale T, Jacob A, Palace J, Maxwell S, Beeson D, Vincent A (2008). Aquaporin-4 antibodies in neuromyelitis optica and longitudinally extensive transverse myelitis. Arch Neurol.

[15] Huda S, Whittam D, Bhojak M, Chamberlain J, Noonan C, Jacob A (2019). Neuromyelitis optica spectrum disorders. Clin Med (Lond).

[16] Rice B, Perera P (2012). Intramedullary spinal neurocysticercosis presenting as Brown-Sequard syndrome. West J Emerg Med.

